# Supplementary material for: Detailed molecular and epigenetic characterization of the pig IPEC-J2 and chicken SL-29 cell lines
Source: iScience. 2023 Feb 20;26(3):106252. doi: 10.1016/j.isci.2023.106252 (PMC10018572; doi:10.1016/j.isci.2023.106252)
Supplement: Data S1. Complete homer output for identified motifs in Pig IPECJ-2, related to Table 2 — Homer motif analysis results for histone modifications H3K4me1, H3K4me3, H3K27ac, and enhancer elements of pig IPECJ2 cell line. P-values >1e-10 are possible false positives. Within each folder (e.g. peak_files_CTCF) are the html files showing the identified motifs when using homer (e.g. homerResults.html). [file mmc2.zip › S5/Pig_IPECJ_2/peak_files_H3K27ac/homerResults/motif5.similar.html]

motif5

## Information for motif5

C
G
T
A
A
G
C
T
A
T
C
G
A
T
C
G
A
G
T
C
A
T
C
G
A
T
C
G
G
T
A
C
A
T
C
G
A
T
C
G
  
Reverse Opposite:  

T
A
G
C
T
A
G
C
C
A
T
G
T
A
G
C
T
A
G
C
A
C
T
G
T
A
G
C
T
A
G
C
C
T
G
A
C
G
A
T
  

|  |  |
| --- | --- |
| p-value: | 1e-104 |
| log p-value: | -2.414e+02 |
| Information Content per bp: | 1.694 |
| Number of Target Sequences with motif | 2318.0 |
| Percentage of Target Sequences with motif | 4.41% |
| Number of Background Sequences with motif | 1413.9 |
| Percentage of Background Sequences with motif | 2.73% |
| Average Position of motif in Targets | 149.8 +/- 89.7bp |
| Average Position of motif in Background | 153.0 +/- 95.6bp |
| Strand Bias (log2 ratio + to - strand density) | -0.0 |
| Multiplicity (# of sites on avg that occur together) | 1.09 |
| Motif File: | file (matrix) reverse opposite |

### Similar de novo motifs found

|  |  |  |  |  |  |  |  |
| --- | --- | --- | --- | --- | --- | --- | --- |
| Rank | Match Score | Redundant Motif | P-value | log P-value | % of Targets | % of Background | Motif file |
| 1 | 0.927 | A C T G A G T C A T G C A C T G T A G C C T A G C G T A G A C T | 1e-88 | -203.891085 | 14.53% | 11.64% | motif file (matrix) |
| 2 | 0.940 | T C A G T A G C T A G C C G T A A C G T A T C G C A T G A G T C A C T G T C A G T A G C A T C G T A C G | 1e-88 | -203.720256 | 4.66% | 3.05% | motif file (matrix) |
| 3 | 0.603 | A C G T G T A C A C T G T A G C T A C G T A C G T C G A A T C G G A C T A T G C | 1e-55 | -128.540129 | 9.32% | 7.44% | motif file (matrix) |
| 4 | 0.620 | G C A T T A G C C T A G T G A C A C T G C T G A A G T C A G T C G A T C G A C T | 1e-46 | -108.106428 | 8.33% | 6.69% | motif file (matrix) |
